# The efficacy of induction chemotherapy or adjuvant chemotherapy added to concurrent chemoradiotherapy in T3-4N0-1M0 nasopharyngeal carcinoma: a propensity score-matched analysis

**DOI:** 10.1080/15384047.2023.2274121

**Published:** 2023-11-15

**Authors:** Qiulu Zhong, Danjing Luo, Xiangde Li, Qinghua Du, Qianfu Liang, Wenqi Liu, Jian Li, Xiaodong Zhu

**Affiliations:** a Department of Radiation Oncology, Guangxi Medical University Cancer Hospital, Nanning, China; b Department of Radiation Oncology, The Second Affiliated Hospital of Guangxi Medical University, Nanning, China; c Department of Radiation Oncology, Wuming Hospital of Guangxi Medical University, Nanning, China

**Keywords:** Nasopharyngeal carcinoma, induction chemotherapy, adjuvant chemotherapy, stage T3-4N0-1, concurrent chemoradiotherapy

## Abstract

This research aimed to assess the effectiveness of combining induction chemotherapy (IC) or adjuvant chemotherapy (AC) with concurrent chemoradiotherapy (CCRT) in patients with T3-4N0-1M0 nasopharyngeal carcinoma (NPC). Before propensity score matching(PSM),we retrospectively collected 457 patients with T3-4N0-1M0 NPC treated with CCRT with or without IC/AC. PSM method selected 285 patients from two cohort(148 in CCRT±IC/AC group,137 in CCRT group). The 3-year overall survival(OS), locoregional relapse-free survival (LRFS) and distant metastasis-free survival (DMFS) were estimated. The median follow-up was 41.03 months(range 2.13–94.67 months). No significant differences in 3 year-OS,LRFS and DMFS between CCRT±IC/AC group and CCRT group.Univariate analysis have shown that induction chemotherapy was significantly associated with 3 year LRFS(hazard ratio[HR] 0.214, 95%confidence interval[CI] 0.053–0.861,*P* = .030).Overall stage(HR 0.260, CI 0.078–0.870, *P* = .029) and T classification (HR 0.260, CI 0.078–0.870, *P* = .029)were significantly associated with OS.Multivariate analysis demonstrated no independent factors were related to 3-year OS,LRFS and DMFS. Subgroup analyses revealed that no significant survival differences in the two groups in patients with T3N1.In terms of T4N1 disease, patients received CCRT±IC/AC had lower 3-year DMFS than those treated with CCRT(90.4% vs 98.7%, *P* = .015). Adding IC or AC to CCRT did not significantly improve the prognosis of T3-4N0-1M0 NPC patients. Patients with T4N1M0 treated with CCRT had better DMFS than those received CCRT±IC/AC.However,more investigations should be confirmed the results.

## Introduction

Nasopharyngeal carcinoma (NPC) is a malignancy with high sensitivity to radiotherapy and is particularly prevalent in regions of southern China, such as Guangdong, Guangxi, Hong Kong, Hu’nan.^1,2^ The majority of patients are diagnosed with locoregionally advanced nasopharyngeal carcinoma(LA-NPC) at the first time to be found.^3^ Patients with T3-4N0-1M0 stage is a unique subgroup in locoregionally advanced NPC which always have better prognosis.^4^ Chemotherapy is widely used as an adjunct to radiotherapy or as induction chemotherapy for LA-NPC, but it is still controversial whether chemotherapy can benefit patients with T3-4N0-1M0 stage disease.^5,6^ Some clinical trials have reported a survival advantage with induction chemotherapy in patients with advanced NPC.For example, In Sun Y’s report, the results showed that TPF is a better induction regimen for LA-NPC which significantly improves failure-free survival.^7^ Xing Lv et al. also pointed out that Lobaplatin-based neoadjuvant chemotherapy followed by concurrent chemoradiotherapy is an non-inferior choice due to fewer toxic effects.^8^ A meta analysis analyzing seven randomized controlled trails revealed that induction chemotherapy followed by concurrent chemoradiotherapy is a better regimen for LA-NPC which can improve prognosis.^9^ Recently, a randomized, phase III clinical trial showed that LA-NPC patients (except T3-4N0) who received IC plus CCRT significantly improved their prognosis.Therefore, IC plus CCRT is a superior regimen recommend for LA-NPC in the latest guidelines,^10^ while other studies have found no significant difference.In a meta analysis, the author found IC could not significantly improve prognosis in LA-NPC.^11^ Since the INT 0099 trial successfully introduced adjuvant chemotherapy(AC) for LA-NPC in 1998,^12^ a variety of chemoradiotherapy schedules have been carried out to investigate the efficacy of AC in LA-NPC.Recently, a phase 3 clinical trail from Sun Yat-sen University Cancer Center found that metronomic capecitabine is a superior regimen for improving prognosis in LA-NPC.^13^ However, several clinical trails from endemic areas revealed that adding AC could not improved prognosis in LA-NPC.^14,15^ Because patients with T3-4N0-1M0 are often excluded in most studies,^16,17^ the efficacy of IC or AC combine with CCRT could not be fully evaluated in this subgroup of patients.Therefore, more studies are needed to investigate the efficacy and safety of chemotherapy in T3-4N0-1M0 stage NPC.In recent years, several clinical trials have been carried out to evaluate the efficacy and safety of various chemotherapy regimens in patients with T3-4N0-1M0 stage NPC. For example, a randomized trial conducted in China showed that induction chemotherapy followed by concurrent chemoradiotherapy may improve OS in patients with T3-4N0-1M0 NPC in the region of Northwest China.^18^ Another study by Li-Rong Wu found that neoadjuvant chemotherapy or adjuvant chemotherapy plus concurrent chemotherapy could not improve the prognosis in patients with T3-4N0-1M0 NPC.^19^ But research about T3-4N0-1M0 NPC subgroup is still limited.Therefore, we retrospectively analyzed patients with T3-4N0-1 NPC who received CCRT with or without IC/AC in the IMRT era. Propensity score matching(PSM) method was used to simulate randomized trials and to reduce potential bias.^20^

## Methods and materials

### Patients

A total of 457 NPC patients with stage T3-4N0-1(AJCC 7th edition) received CCRT with or without IC or AC were retrospectively reviewed in The First Affiliated hospital of Guangxi Medical University and The Second Affiliated hospital of Guangxi Medical University from January 2012 to December 2020. The inclusion criteria were as follows:(1)Age between 16 and 80 years old;(2)T3-4N0-1M0 disease staged by using the 7^th^ edition of the American Joint Committee on Cancer(AJCC) TNM staging system; (3)ECOG performance score ≤ 2;(4)Pathological type was Non-keratinizing undifferentiated carcinoma or differentiated carcinoma;(5)Patients treated with Intensity-modulated radiotherapy(IMRT);(6)Complete follow-up data and clinical data were available.Exclusion criteria included patients with double cancer, or patients with distant metastasis before treatment, patients who had serious heart, lung, kidney or liver diseases and incomplete follow-up data.All clinical records including head and neck magnetic resonance imaging(MRI),chest computerized tomography (CT), abdominal ultrasound, nasopharyngeal fiber optic endoscopy and bone scan were reviewed.

### Radiotherapy

All enrolled patients were treated with one daily fraction of intensity-modulated radiotherapy(IMRT) for 5 days per week.Target volumes and organ at risks(OARs) were delineated based on the International Commission on Radiation Units and Measurements Reports 50 and 62. Gross tumor volumes included nasopharynx GTV(GTVnx) and the cervical lymph nodes GTV(GTVnd).The high risk clinical target volumes were contoured as CTV1(CTV1=GTVnx plus 6-8 mm margins and the whole nasopharynx).The low risk clinical target volumes was defined as the CTV1 plus a 5-10 mm margin to contain the low risk regions including parapharyngeal space, sphenoid sinus, retropharyngeal nodal regions, posterior parts of the nasal cavity, clivus, pterygopalatine fossae, pterygoid fossae, and the selective neck area.The prescribed doses were 68-74 Gy to the GTVnx,62-70 Gy to the GTVnd,60-66 Gy to the CTV1 and 50-56 Gy to the CTV2 for 30–35 fractions.

### Chemotherapy

The induction chemotherapy(IC) schemes including TPF regimen: docetaxel (60 mg/m2,day 1),cisplatin (60 mg/m2, day 1) and fluorouracil (600 mg/m2, day 1 to day 5, or continuous intravenous infusion for 120 hours) every three weeks for two to three cycles;TP regimen:docetaxel (75 mg/m2,day 1),cisplatin (75 mg/m2,day 1) every three weeks for two to three cycles;PF regimen:cisplatin (80 mg/m2, day 1)and fluorouracil (750 mg/m2, day 1 to day 5 or continuous intravenous infusion for 120 hours) every three weeks for two to three cycles;GP regimen:Gemcitabine (1 g/m2, day 1) and cisplatin (80 mg/m2, day 1) every three weeks for two to three cycles.

The CCRT regimens:Cisplatin alone :80-100 mg/m2 every three weeks for two to three cycles, or 30-40 mg/m2 weekly for four to six cycles.

The adjuvant chemotherapy regimens:TPF regimen: docetaxel (60 mg/m2, day 1),cisplatin (60 mg/m2, day 1) and fluorouracil (600 mg/m2, day 1 to day 5 or continuous intravenous infusion for 120 hours) every three weeks for two to three cycles;TP scheme:docetaxel (75 mg/m2,day 1),cisplatin (75 mg/m2,day 1) every three weeks for two to three cycles;PF scheme:cisplatin (80 mg/m2, day 1)and fluorouracil (750 mg/m2, day 1 to day 5 or continuous intravenous infusion for 120 hours) every three weeks for two to three cycles;GP scheme:Gemcitabine (1 g/m2, day 1) and cisplatin (80 mg/m2, day 1) every three weeks for two to three cycles.

### Follow-up

The follow-up time was measured from the day of first to receive treatment to the day of death or the last time to follow-up. The patients underwent EB virus DNA copies, complete blood cell count, liver and kidney functions, physical examination, fiber optic endoscopy, magnetic resonance imaging(MRI)scans for nasopharynx and neck, chest computerized tomography(CT), abdominal ultrasound or CT of abdominal and whole body bone scan every 6 months.Patients assessed for every three months in the first two years,then every six months for three to five months, and 12 months thereafter. The endpoints were 3-year overall survival(OS), locoregional relapse-free survival (LRFS) and distant metastasis-free survival(DMFS).These end points were calculated from the date of the first to receive therapy to the date of death or the last to follow-up, first locoregional relapse, distant metastasis.

## Statistical analysis

All the statistical analysis were performed to use R(version 3.6.3, available at www.r-project.org) and SPSS 22.0(IBM Corp).We used the propensity score matching (PSM) method to match the patients between the two groups(CCRT±IC/AC and CCRT) in a ratio of 1:1.The categorical variables were compared by χ2 test or Fisher’s exact test.Continuous variables were examined by using independent-samples t-test.The Kaplan-Meier method and log-rank test were used to compare survival.Univariate and Multivariate analyses were performed by using the Cox proportional hazards model to identify the independent prognostic factors.Statistical significance was determined by two-tailed P-values <.05.

## Results

### Patients characteristics

Initially, there were 417 patients who met the inclusion criteria.However, before performing PSM,the two groups were found to be imbalanced in terms of T classification, overall stage and target molecular therapy (P < .01,P < .01,P = .006,respectively).After applying the PSM method, a total of 285 patients were selected for further analysis.Within the PSM cohort, there were 148 patients in the CCRT±IC/AC group and 137 patients in the CCRT group. The median age of the patients was 47 years old(range 28 to 69).The distribution of patients in CCRT±IC/AC group and in CCRT group was balanced (Table 1).

**Table 1.** Baseline patient characteristics in the cohort before and after propensity score matching.

	Treatment Methods					
	pre-PSM post-PSM			pre-PSM post-PSM		
Variables	ICCRT±IC/AC group(*n* = 211)	CCRT group (*n* = 206)	*P* value	ICCRT±IC/AC group(*n* = 148)	CCRT group (*n* = 137)	*P* value
Age	19y-68y(48y)	27y-75y(48y)		30y-69y(47y)	28y-69y(47y)	
Age group			.28			.51
>60	28(13.3)	36(17.5)		20(13.5)	15(10.9)	
≤60	183(86.7)	170(82.5)		128(86.5)	122(89.1)	
Gender			.419			.082
Male	156(73.9)	145(70.4)		116(78.4)	95(69.3)	
Female	55(26.1)	61(29.6)		32(21.6)	42(30.7)	
ECOG			.68			.681
0–1	189(89.6)	187(90.8)		137(92.6)	125(91.2)	
=2	22(10.4)	19(9.2)		11(7.4)	12(8.8)	
Pathologic type			.94			1.000
Non-keratinizing undifferentiated carcinoma	193(91.5)	188(91.3)		146(98.6)	135(98.5)	
Non-keratinizing differentiated carcinoma	18(8.5)	18(8.7)		2(1.4)	2(1.5)	
Overall stage			<.001			.065
III	60(28.4)	95(46.1)		45(30.4)	56(40.9)	
IV	151(71.6)	111(53.9)		103(69.6)	81(59.1)	
T Classification			<.001			.065
T3	95(46.1)	60(28.4)		45(30.4)	56(40.9)	
T4	111(53.9)	151(71.6)		103(69.6)	81(59.1)	
N Classification			.402			.32
N0	28(13.6)	23(10.9)		3(2)	6(4.4)	
N1	178(86.4)	188(89.1)		145(98)	131(95.6)	
Target molecular therapy						.61
Yes	3(1.4)	14(6.8)	.006	1(0.7)	2(1.5)	
No	208(98.6)	192(93.2)		147(99.3)	135(98.5)	

### Survival outcomes

**Figure 1.**
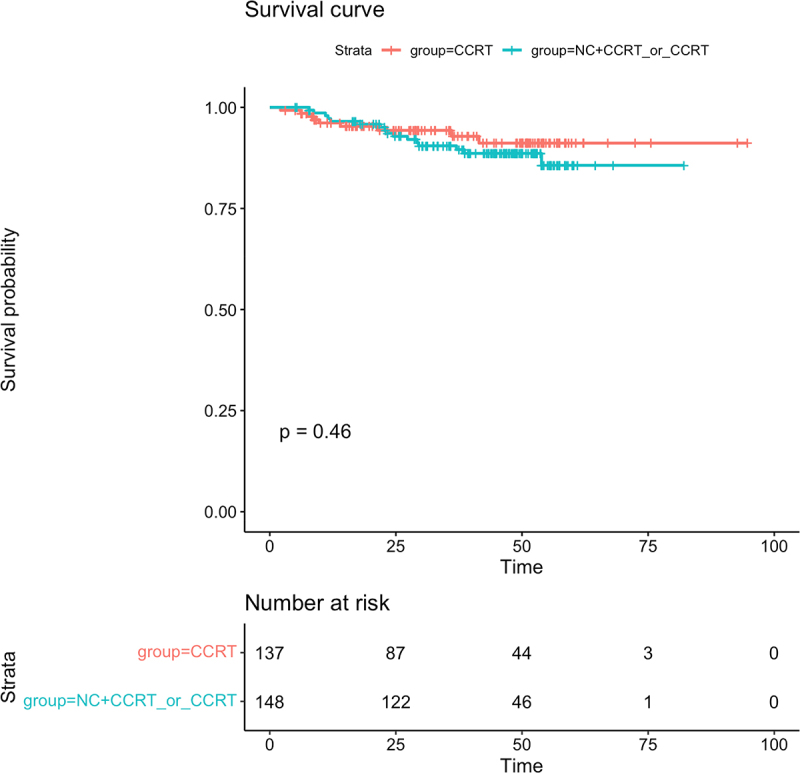
The Kaplan – Meier survival curve for overall survival in T3-4N0-1M0 NPC patients between CCRT±IC/AC group and CCRT group. There was no significant difference in 3-year overall survival between CCRT±IC/AC group and CCRT group(90.5% vs 94.3%,*P* = .46).

**Figure 2.**
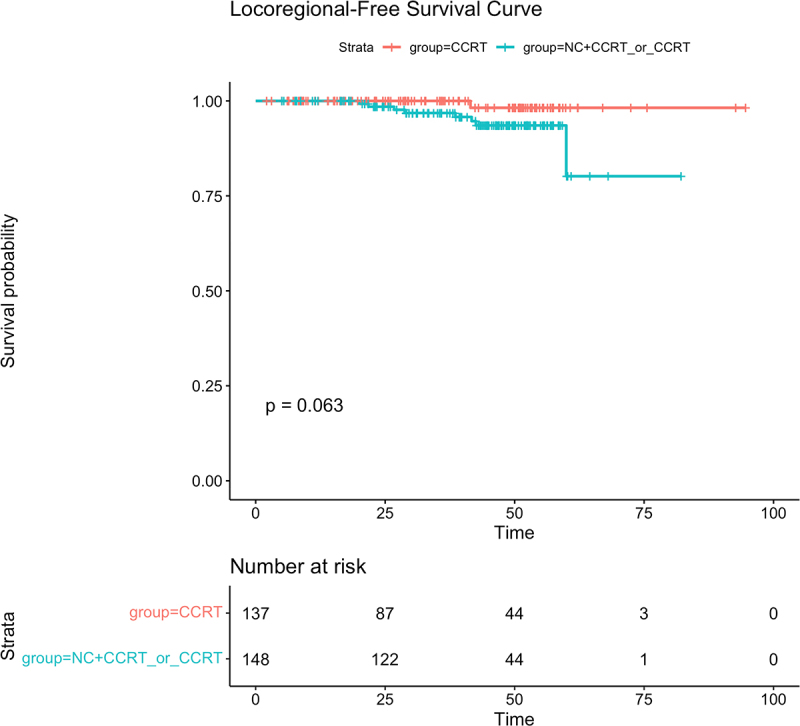
The Kaplan – Meier survival curve for locoregional relapse-free survival in T3-4N0-1M0 NPC patients between CCRT±IC/AC group and CCRT group. There was no significant difference in 3-year overall survival between CCRT±IC/AC group and CCRT group (96.8% vs 100%,*P* = .063).

**Figure 3.**
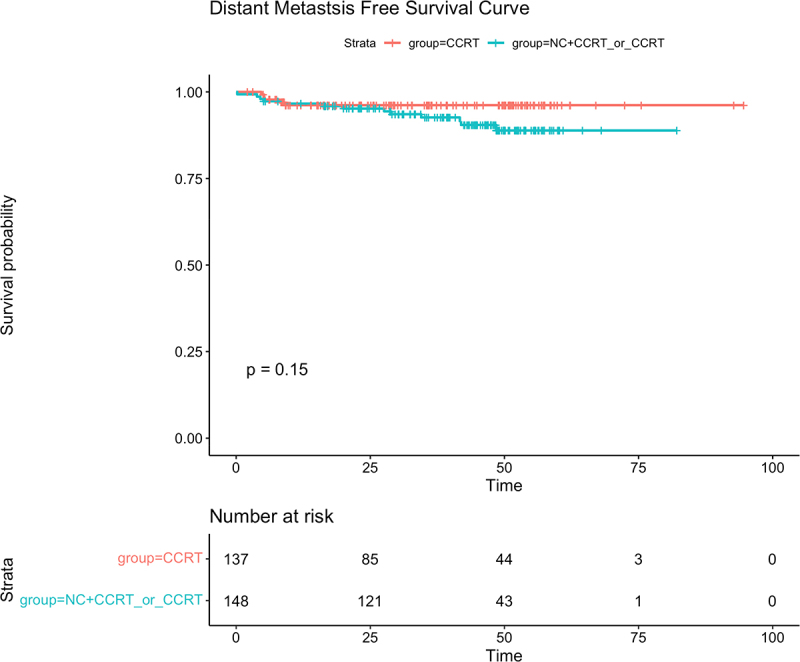
The Kaplan – Meier survival curve for distant failure-free survival in T3-4N0-1M0 NPC patients between CCRT±IC/AC group and CCRT group. There was no significant difference in 3-year overall survival between CCRT±IC/AC group and CCRT group (93.6% vs 96.2%,*P* = .15).

The median follow-up time was 41.03 months(range:2.13–94.67 months).Up to the last follow-up, a total of 25 patients had died,including 18 cases of distant metastasis, 9 cases of regional relapse, and 3 cases of both locoregional relapse and distant metastasis within the entire group.More than half of the patients (16/25,64%) in the CCRT±IC/AC group died, compared with nine of 25 (36%) patients in the CCRT group.Relapse occurred in 8 of 9 (88.9%) patients in the CCRT±IC/AC group and in 1 of 9 (11.1%) patients in the CCRT group. In addition,13 of 18 (72.2%) patients experienced distant metastasis in the CCRT±IC/AC group, and only a small percentage of patients had distant metastasis in the CCRT group (5/18, 27.8%). Of the CCRT±IC/AC cohort, the 3-year OS,LRFS,DMFS were 90.5%,96.8% and 93.6%,respectively, While in the CCRT group,the 3-year OS,LRFS and DMFS were 94.3%,100% and 96.2%,respectively. No significant differences were observed in 3-year OS,LRFS and DMFS between the two groups(OS:90.5% vs 94.3%,*P* = .46, Figure 1; LRFS:96.8% vs 100%, *P* = .063, Figure 2; DMFS:93.6% vs 96.2%,*P* = .15,respectively, Figure 3). Univariate analysis (Table 2) revealed that only the induction chemotherapy was significantly associated with LRFS(hazard ratio[HR] 0.214, 95%confidence interval[CI] 0.053–0.861,*P* = .030). In addition, Overall stage(HR 0.260, CI 0.078–0.870, *P* = .029) and T classification (HR 0.260, CI 0.078–0.870, *P* = .029) were significantly associated with OS. Multivariate analysis (Table 2) was performed, and no significant differences were found in age, gender, overall stage, ECOG, induction chemotherapy, pathological type, T classification,N classification, adjuvant chemotherapy, target molecular treatment and treatment method.

**Table 2.** Univariate and multivariate analyses for OS, LRFS and DMFS based on the clinical characteristics.

	Univariate Analyses									multivariate analyses								
	LRFS			OS			DMFS			LRFS			OS			DMFS		
variable	HR	95%CI	P value	HR	95%CI	P value	HR	95%CI	P value	HR	95%CI	P value	HR	95%CI	P value	HR	95%CI	P value
Gender female vs male	1.181	0.243–5.748	.837	1.308	0.491–3.489	.591	1.679	0.486–5.801	.413	0.93	0.18–4.7	.93	1.2	0.42–3.1	.780	1.4	0.39–4.8	.63
Age >60 vs ≤ 60	0.997	0.124–7.992	.998	0.426	0.170–1.067	.069	0.486	0.16–1.478	.204	1.1	0.13–9.2	.94	0.53	0.2–1.4	.200	0.53	0.17–1.7	.28
Overall stage IV vs III	0.245	0.030–1.973	.186	0.260	0.078–0.87	.029	0.763	0.271–2.147	.609	NA	NA-NA	NA	NA	NA-NA	NA	NA	NA-NA	NA
ECOG 2 vs 0–1	83496489	0-nf	.998	1.847	0.250–13.66	.548	1.341	0.178–10.09	.775	8.8e+07	0-Inf	1.00	1.1	0.13–8.7	.950	1.1	0.13–9.1	.93
Induction chemotherapy Yes vs No	0.214	0.053–0.861	.030	0.531	0.241–1.172	.117	0.505	0.197–1.282	.15	0.52	0.069–3.9	.53	0.84	0.18–4	.830	0.72	0.12–4.3	.72
Pathologic type Non-keratinizing differentiated carcinoma vs Non-keratinizing undifferentiated carcinoma	9150669	0-Inf	.998	0.359	0.048–2.656	.316	9115011	0-Inf	.998	1.5e+08	0-Inf	1.00	0.6	0.077–4.7	.630	3.1e+07	0-Inf	1.00
T classification T4 vs T3	0.245	0.030–1.973	.186	0.260	0.078–0.87	.029	0.763	0.271–2.147	.609	0.33	0.04–2.8	.31	0.32	0.093–1.1	.071	0.92	0.31–2.7	.88
N classification N1 vs N0	3.7e-08	0-Inf	.998	3.894e-08	0-Inf	.997	3.916e-08	0-Inf	.998	2.8e-08	0-Inf	1.00	9.8e-08	0-Inf	1.00	5.4e-08	0-Inf	1.00
TN stage T4N1 vs T4N0 T4N1 vs T3N1 T4N1 vs T3N0	NA 0.286 2.604e-08	NA 0.036–2.295 0-Inf	NA .239 .998	NA 0.291 2.771e-08	NA-NA 0.087–0.973 0-Inf	NA .045 .998	NA 0.851 3.725e-08	NA-NA 0.302–2.394 0-Inf	NA .760 .998	NA NA NA	NA-NA NA-NA NA-NA	NA NA NA	NA NA NA	NA-NA NA-NA NA-NA	NA NA NA	NA NA NA	NA-NA NA-NA NA-NA	NA NA NA
Adjuvant chemotherapy Yes vs No	0.960	0.240–3.840	.954	1.395	0.556–3.499	.478	0.846	0.317–2.256	.738	0.92	0.15–5.6	.93	1.1	0.25–4.7	.910	0.86	0.16–4.6	.86
Target molecular treatment Yes vs No	9088811	0-Inf	.999	9069146	0-Inf	.998	9062805	0-Inf	.998	5.2e+07	0-Inf	1.00	3.5e+07	0-Inf	1.00	2.3e+07	0-Inf	1.00
Treatment method IC or AC+CCRT_vs CCRT	5.756	0.717–46.22	.100	1.36	0.600–3.082	.462	2.115	0.752–5.945	.155	3.6	0.22–60	.37	NA	NA-NA	NA	1.5	0.21–11	.68

### Subgroup analysis

**Figure 4.**
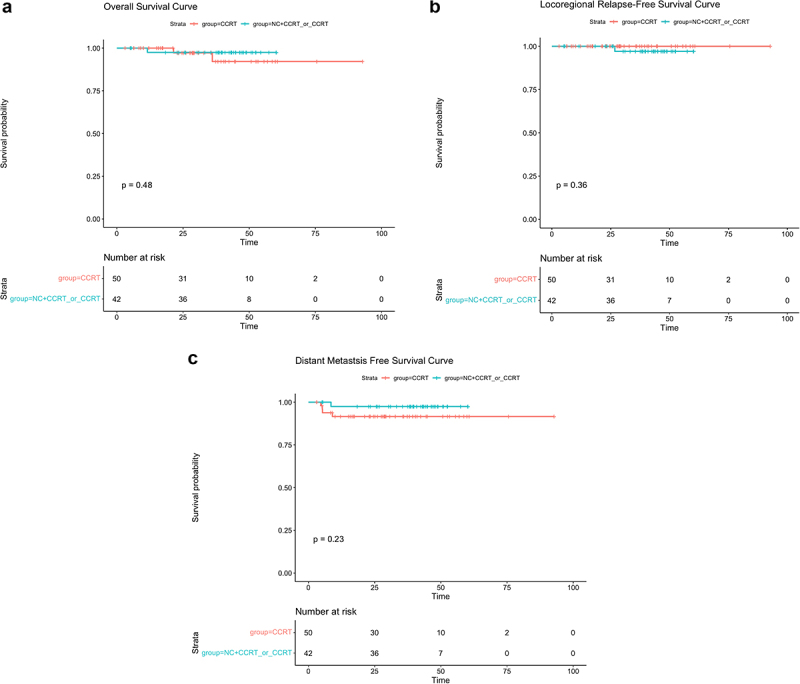
The Kaplan – Meier survival curve for the T3N1M0 subgroup: (a) overall survival, (b) locoregional relapse-free survival, (c) distant metastasis-free survival. There were no significant differences in the 3-year OS, LRFS and DMFS between CCRT±IC/AC group and in CCRT group

**Figure 5.**
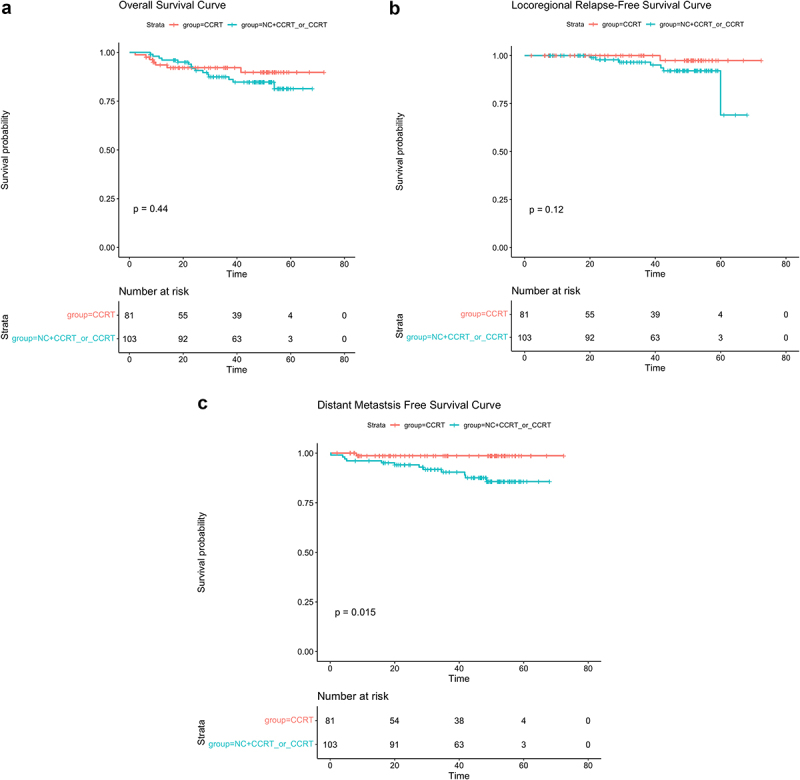
The Kaplan – Meier survival curve for the T4N1M0 subgroup: (a) overall survival, (b) locoregional relapse-free survival, (c) distant metastasis-free survival.Survival.The 3-year OS and DMFS was similar in CCRT±IC/AC group and in CCRT group, but the 3-year DMFS were remarkably higher in CCRT group than in CCRT±IC/AC group.

Among the inclusion patients, There were 9 patients with T3N0 tumor, 92 patients with T3N1 disease, and 184 patients with T4N1disease.Because patients with T3N0 disease was too small to analyze, so we only analyzed patients with T3N1 and patients with T4N1 subgroups.For patients with T3N1 disease, the 3-year OS, LRFS and DMFS were similar in CCRT±IC/AC group and in CCRT group (OS: 97.5% vs 92.1%, *P* = .48, Figure 4(a); LRFS: 97.1% vs 100%, *P* = .36, Figure 4(b); DMFS: 97.5% vs 91.6%, *P* = .23, Figure 4(c)). In terms of T4N1 patients, the 3-year OS and LRFS was similar in CCRT±IC/AC group and in CCRT group (OS:87.4% vs 92.1%, *P* = .44, Figure 5(a); LRFS: 96.6% vs 97.4%, *P* = .12, Figure 5(b), but the 3-year DMFS were remarkably higher in CCRT group than in CCRT±IC/AC group (98.7% vs 90.4%, *P* = .015, Figure 5(c)).

## Discussion

Our study found that patients with T3-4N0-1M0 treated with CCRT alone or CCRT±IC/AC did not show improvement in3-year OS, LRFS and DMFS. Subgroup analysis revealed that the two treatment groups showed no significant difference for patients with T3N1M0 NPC. However, for T4N1M0 NPC, the addition of IC or AC to CCRT did not improve survival but reduced the DMFS. As expected, IC or AC did not show any survival benefits for patients with T3-4N0-1M0 NPC.

Many studies have demonstrated the value of IC combined with CCRT in LA-NPC (Table 3). Sun et al.^22^ demonstrated that IC+CCRT was a tolerable treatment method for LA-NPC, with the potential to improve failure-free survival.Yang et al.^23^ also carried out a randomized phase III clinical trial to compare IC+CCRT with CCRT for LA-NPC.The outcomes showed that IC+CCRT significantly improved survival.They suggested that cisplatin and fluorouracil as neoadjuvant chemotherapy followed by CCRT significantly improved survival benefit. In zhang’s^16^ research, they prospectively collected 480 NPC cases to evaluate the efficacy of gemcitabine and cisplatin as an induction chemotherapy regimen for LA-NPC.Several other studies reached the same conclusion. It should be noted that patients with T3-4N0-1M0 NPC have often been ruled out in these phase III RCTs (Table 3) Therefore, the optimal treatment for these subgroups remains controversial.

**Table 3.** Related-references about IC plus CCRT in LA-NPC.

Author	Year	Study design	Patients	Groups	Result	median follow-up	IC regime
Sun	2016^7^	Prospective3	stage III-IVB (except T3-4N0)	IC+CCRT vs CCRT	3-year FFS 80% vs 72%,*p* = .034	45months	TPF
Frikha	2018^21^	Prospective3	stage III-IVB (except T3-4N0)	IC+CCRT vs CCRT	3-year PFS 73.9% vs 57.2%, *P* = .042 3-years OS 86.3% vs 68.9% *P* = .05	43.1months	TPF
Hong	2018^22^	Prospective3	stage IVA-IVB	IC+CCRT vs CCRT	5-year DFS 61% vs 50%, *P* = .0264	72months	MEPFL
Li	2019^17^	Prospective3	stage III-IVB (except T3-4N0)	IC+CCRT vs CCRT	5-year FFS 77.4% vs. 66.4%, *p* = .019 5-yearOS 85.6% vs. 77.7%, *p* = .042 5-year DFFS 88% vs. 79.8%, *p* = .030 5-yearLFFS 90.7% vs. 83.8%, *p* = .044	71.5 months	TPF
Yang	2019^23^	Prospective3	stage III-IVB (except T3N0–1)	IC+CCRT vs CCRT	5-year DFS 73.4% vs 63.1%, *p* = .007 5-year DMFS 82.8% vs 73.1%, *p* = .014 5-year OS 80.8% vs 76.8%, *p* = .040	82.6 months	PF
Zhang	2022^24^	Prospective3	stage III-IVB (except T3-4N0)	IC+CCRT vs CCRT	5-year OS 87.9% vs 78.8%, *P* = .001	69.8months	GP

Of course, some researchers have begun to investigate this unique group of people. Li et al.’s research^25^ focused on 362 patients diagnosed with T3-4N0 NPC. With a 95-month follow-up time, T3N0M0 patients in the CCRT group had a better prognosis than those in the IC+CCRT group, However, in T4N0M0 patients, both management approaches had a similar therapeutic effect. Lan et al.^26^ reported no significant difference in patients with T3N0–1 disease, IC combined with CCRT was not linked with improved prognosis. But EBV-DNA (4000 copies/mL as cutoff)was associated with 4-years DMFS. Xu et al.^27^ retrospectively^28^ collected NPC patients categorized into T3N0, T3N1, T4N0 subgroups. The results indicated that selecting IC-sensitive patients was feasible. Patients with high- risk (EBV-DNA ≥2,000 copies/mL, male)T3N1 NPC had a better prognosis when treated with IC+CCRT.T3N0 and T4N0 need more further investigations in future IC-related studies.Although the previous study examined this type of special stage population and found no unified conclusion, we attempted to add adjuvant chemotherapy to see if it could increase benefits. Unfortunately, the findings revealed no benefits. Lee et al.^29^ demonstrated a long-term follow-up study that there was no significant difference between CCRT followed by adjuvant chemotherapy and CCRT alone, which is consistent with the results of our studies on adjuvant chemotherapy.However, Mu et al.^30^ compared the efficacy of IC+CCRT with CCRT+AC, and the results revealed that patients who received IC+CCRT had a better prognosis and experienced fewer side-effects. In our research, the subgroup analysis revealed that no significantly difference in T4N1 NPC patients, but for T3N1 NPC patients, IC or AC added to CCRT did not improve survival but reduced the DMFS. We considered that IC or AC reduced the tolerance of CCRT,more chemotherapy-related toxic and side effects exceed the survival benefits of chemotherapy. Moreover, we need to consider whether the statistical survival benefit of induction chemotherapy was offset by enrolling both induction chemotherapy and adjuvant chemotherapy patients. This is an important aspect that should be taken into account.Besides, as we mentioned above, previous studies have shown that induction chemotherapy can improve the prognosis of LA-NPC, especially for N2–3 NPC patients.However, the efficacy of induction chemotherapy in patients with T3-4N0 and N1 disease remains controversial. Nonetheless, our research is still noteworthy.Our study is a multicenter retrospective study aimed at evaluating the effectiveness of IC or AC added to CCRT in T3-4N0-1M0 NPC patients, and we used PSM method to minimize the potential bias in the research.

Our study still had several limitations. First, the number of T3N0 and T4N0 NPC patients was too small, we could not assess the effectiveness of IC or AC in this subgroup of patients. Second, the various regimens of IC or AC in the cohort may have influenced the results. In the present study, the regimens of IC included GP,TPF,TP and PF,whereas AC regimens also included GP,TPF,TP and PF. Third, we did not evaluate the plasma pre-EBV-DNA, which is closely related to the prognosis of NPC.Finally, we did not evaluate the side-effects in this study. Nevertheless, our study is noteworthy because the efficacy of IC or AC in subgroups like T3N0,T3N1,T4N0 and T4N1 is still limited, and more data are needed to confirm their effectiveness.

In conclusion, the addition of IC or AC to CCRT did not provide survival benefits for T3-4N0-1 NPC patients. Subgroup analysis revealed that patients with T4N1 who treated with CCRT alone had higher DMFS than those who received IC+CCRT or CCRT+AC. However, to confirm these conclusion, more large prospective and randomized controlled clinical trials are required.
